# Classification of premalignant pancreatic cancer mass-spectrometry data using decision tree ensembles

**DOI:** 10.1186/1471-2105-9-275

**Published:** 2008-06-11

**Authors:** Guangtao Ge, G William Wong

**Affiliations:** 1 Department of Computer Science, Tufts University, Medford, MA, 02155, USA; 2 Department of Physiology and the Center for Metabolism and Obesity Research, Johns Hopkins University School of Medicine, Baltimore, MD, 21205, USA

## Abstract

**Background:**

Pancreatic cancer is the fourth leading cause of cancer death in the United States. Consequently, identification of clinically relevant biomarkers for the early detection of this cancer type is urgently needed. In recent years, proteomics profiling techniques combined with various data analysis methods have been successfully used to gain critical insights into processes and mechanisms underlying pathologic conditions, particularly as they relate to cancer. However, the high dimensionality of proteomics data combined with their relatively small sample sizes poses a significant challenge to current data mining methodology where many of the standard methods cannot be applied directly. Here, we propose a novel methodological framework using machine learning method, in which decision tree based classifier ensembles coupled with feature selection methods, is applied to proteomics data generated from premalignant pancreatic cancer.

**Results:**

This study explores the utility of three different feature selection schemas (Student *t *test, Wilcoxon rank sum test and genetic algorithm) to reduce the high dimensionality of a pancreatic cancer proteomic dataset. Using the top features selected from each method, we compared the prediction performances of a single decision tree algorithm C4.5 with six different decision-tree based classifier ensembles (Random forest, Stacked generalization, Bagging, Adaboost, Logitboost and Multiboost). We show that ensemble classifiers always outperform single decision tree classifier in having greater accuracies and smaller prediction errors when applied to a pancreatic cancer proteomics dataset.

**Conclusion:**

In our cross validation framework, classifier ensembles generally have better classification accuracies compared to that of a single decision tree when applied to a pancreatic cancer proteomic dataset, thus suggesting its utility in future proteomics data analysis. Additionally, the use of feature selection method allows us to select biomarkers with potentially important roles in cancer development, therefore highlighting the validity of this method.

## Background

Pancreatic cancer is one of the most lethal types of cancer. In United States, there are ~30,000 new cases being diagnosed each year. The mortality rate of pancreatic cancer patients is approaching 100%. Only 4% of the patients survive 5 years or more after being diagnosed. The grim statistics of pancreatic cancer necessitates the urgent development of methods to facilitate their early detection and prevention [1]. Despite the advancement of our knowledge in recent years regarding the pathophysiology of pancreatic cancer [2,3], we still lack an effective method to diagnose this cancer type early enough to impact the treatment outcomes.

Recently, there has been substantial interests in applying proteomics technology to identify clinically useful biomarkers for early-stage pancreatic cancer [4-11]. In a more general sense, many investigators have applied proteomics technology and data mining methods to identify serum proteomic patterns that can distinguish normal from cancer samples. Examples of these include ovarian cancer [12-17], breast cancer [18,19], prostate cancer [20-22], lung cancer [23], brain tumors [24], and head and neck cancer [25].

One of the major challenges for proteomic profiling is the analysis and mining of biologically useful information from the enormous dataset. Due to the high dimensionality of proteomics dataset and their often small sample sizes, non-classical statistical methods for data analysis need to be employed. Therefore, various machine learning classification algorithms have been applied to proteomics data analysis. These include the use of decision tree [26,27], boosted decision tree [28], random forest [29], nearest centroid [30], Bayesian neural network [31], self-organizing map [32], support vector machine [33,34], linear and quadratic discriminant analysis [35] and meta-learners [36,37]. However, there are limitations regarding these studies [38-41]. These include the lack of efficient procedure for biomarker selection and the inability to cope with data noise. More importantly, most of these classification methods were constructed based on a single classifier derived from a single training process. They are not robust enough to handle the great variance inherent in the proteomics data. Thus, a more general machine learning method is needed to overcome these challenges.

Here, we present a computational method to analyze a proteomics dataset obtained from premalignant pancreatic cancer using decision tree based classifier ensembles coupled with three feature selection schemas and show that classifier ensembles always have better performances compared to a single decision tree and other models.

## Results

**Figure 1:**
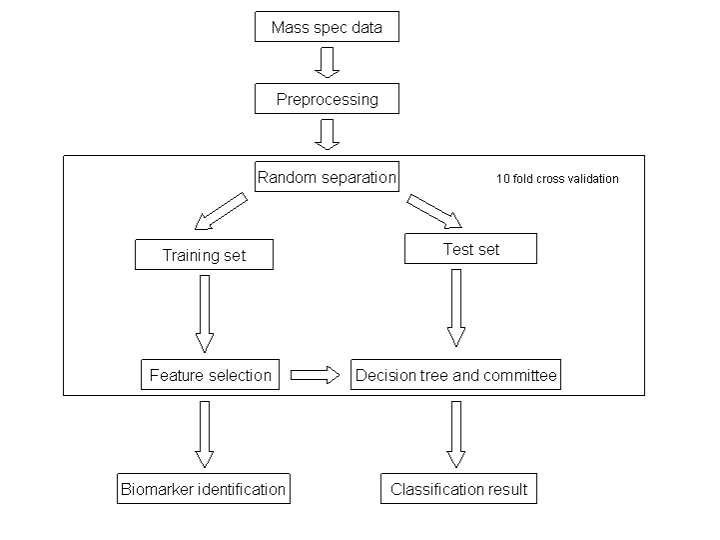
**Computation procedure used in this study**. In each round of 10 fold cross-validation, the whole dataset was randomly separated into training set and test set. Features that significantly differentiate the control class from the disease class are selected using training set only. Then test sets are classified by decision tree and ensembles using these features. Mass spec: mass spectrometry.

The premalignant pancreatic cancer mass spectrometry dataset used in this study include 181 samples. Of the 181 samples, 101 are control serum samples and 80 are PanIN (pancreatic intraepithelial neoplasias) samples. Control samples are referred to as normal cases while PanIN samples as disease cases. The complete computational procedure used in this study is shown in Figure. 1. After preprocessing, we ran our processed data through a 10 fold cross-validation framework. In each round of the cross validation, 90% of the data were selected randomly as training set to build classifier. Three feature selection methods were applied to select top features (mass to charge ratios, *m/z*) from the training set only. Classifiers were then tested on the rest of the 10% data using those selected features. The performances of various classifiers were also compared.

### Data preprocessing

**Figure 2:**
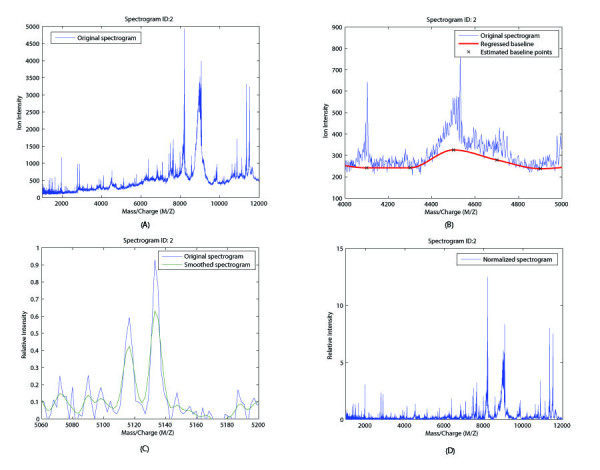
**Data preprocessing result**. Spectrogram ID 2 is used as an example of data preprocessing procedure. (A) Original spectrogram without any processing. The maximum *m*/*z *ratio is 11922.91 and the minimum *m*/*z *ratio is 800. (B) Original spectrogram and adjusted baseline. (C) Noise reduction using Gaussian kernel smoothing. (D) Normalization using the area under the curve (AUC).

To compensate for systematic differences due to sample loadings and instrument errors, raw proteomics data have to be preprocessed before any feature selection method and classification algorithm can be applied. Three major preprocessing procedures were applied to our dataset: baseline adjustment, normalization and kernel smoothing. Using one specific spectrogram as an example, the effects of these processing operations on the raw data are shown in Figure. 2. The original spectrograms consist of 6771 different *m/z *ratios and they range from 800 to 11992.91 in their values (Figure. 2A). The spectrogram baselines were adjusted based on the group median (Figure. 2B). All data points were smoothed by substituting their values with the weighted average of 5 value points on each side using a Gaussian kernel (Figure. 2C). Using the area under each spectrogram curve (AUG), all spectrograms were normalized and rescaled such that their maximum values equal to 100 (Figure. 2D).

### Biomarker identification

In general, classifiers cannot successfully handle high dimensional dataset generated from proteomics experiments. To overcome this problem, we used three feature selection schemes (Student *t *test, Wilcoxon rank sum test and genetic algorithm) to reduce the dimensionality of the dataset to a manageable number. Table. 1 lists all the top 10 features generated from each round of two-sample homoscedastic *t *test. These features are ranked based on their *p*-values that correspond to the probability of their observed differences in mean intensity between control and disease group being significant. Interestingly, several features (*m/z *ratios) such as 5798.9 and 5801.2 were repeatedly selected in our 10 rounds of cross validation analyses despite the fact that the training dataset is randomly selected from the whole dataset each time. Regardless of how the data is partitioned, highly significant differences in peptides' *m*/*z *intensity between control and disease samples can and will likely be selected each time. Thus, these *m/z *ratios are considered potentially good biomarkers for disease identification. The use of randomly selected training data provides greater confidence to our results.

**Table 1:** Top ten features (*m*/*z *ratio) selected by Student *t *test method in our 10 fold cross validation.

Rank	Round 1	Round 2	Round 3	Round 4	Round 5	Round 6	Round 7	Round 8	Round 9	Round 10	Most Frequent
1	5798.9	5798.9	5819.8	5798.9	5819.8	5819.8	5798.9	5798.9	11477	5798.9	5798.9
2	5801.2	5819.8	5822.1	5801.2	5822.1	5822.1	5801.2	11541	11774	5801.2	5801.2
3	5819.8	5801.2	5798.9	5819.8	5798.9	5798.9	5819.8	11592	11472	11592	5819.8
4	5796.5	5822.1	5801.2	5822.1	5801.2	11592	5822.1	5801.2	5798.9	11597	11541
5	5822.1	11541	11592	5829.1	11770	11597	11541	11537	11481	11587	11592
6	11422	11592	11597	5831.4	11541	11587	5831.4	11546	5819.8	11541	5822.1
7	5817.4	11546	11541	11592	11597	5801.2	11592	11597	11770	11601	11597
8	11774	11587	11601	5803.5	11592	11541	11546	11774	11514	5819.8	11546
9	11541	11537	11546	11541	11601	11643	5829.1	11587	11509	11546	11601
10	11426	11569	11639	5796.5	11606	11601	11597	11601	5822.1	11606	11587

Rank is determined by the probability of the two means between disease and control groups in the training set being significantly different. *m*/*z *ratios with smaller probability ranks higher. Most frequent features are determined by the frequency of each feature appears in the top 10 list in these ten runs and ranked by their frequency.

While *t *test assumes that the feature values from two different classes follow normal distributions. In reality, this is often not the case. Therefore, we explored the possibility of using a nonparametric (distribution-free) test to select our top features. The top 10 features selected from Wilcoxon nonparametric rank test are presented in Table 2. Similar to *t *test, some of the *m/z *ratios such as 5798.9 and 5801.2 were also selected frequently. Features that are repeatedly selected from independent runs suggest that those features play important roles in discriminating between normal and disease classes.

**Table 2:** Top ten features (*m*/*z *ratio) selected by Wilcoxon rank test method in our 10 fold cross validation.

Rank	Round 1	Round 2	Round 3	Round 4	Round 5	Round 6	Round 7	Round 8	Round 9	Round 10	Most Frequent
1	5798.9	5798.9	4941.6	5801.2	5822.1	5819.8	5801.2	5798.9	4941.6	5801.2	5798.9
2	5801.2	5801.2	5819.8	5798.9	5819.8	5822.1	5798.9	5801.2	11774	5798.9	5801.2
3	4941.6	11472	5822.1	4941.6	5798.9	5798.9	4941.6	11472	5798.9	5796.5	11472
4	5796.5	5819.8	5801.2	5819.8	5801.2	5801.2	5803.5	11477	11770	11472	5819.8
5	5819.8	11477	5798.9	9706.1	11472	11592	5822.1	11774	11477	5803.5	5822.1
6	5822.1	5822.1	4943.6	5822.1	11477	11587	11472	11770	11472	11592	11477
7	4943.6	11468	11592	5803.5	11468	11472	5819.8	11541	5819.8	11541	11541
8	11472	5796.5	11541	5796.5	11770	11541	11477	11537	5822.1	5819.8	4941.6
9	11774	11541	11472	11472	4941.6	11774	5829.1	11468	5801.2	11477	11774
10	11477	11481	11597	9710	11774	5796.5	11541	11481	11481	11468	5796.5

Rank is determined by the probability of the two means between disease and control groups in the training set being significantly different.

To compare with filter feature selection method such as *t *test and Wilcoxon rank test, we also explored the possible utility of a wrapper method, specifically the use of genetic algorithm coupled with linear discriminant analysis. In contrast to *t *test and Wilcoxon rank test in which several features were repeatedly selected, genetic algorithm provided a very different result. As shown in Table 3, features selected from each round are quite different, with no feature being selected more than twice in 10 rounds of cross-validation. One possible reason for this result is that the initial population size used by the genetic algorithm was small and that it was randomly selected from the training set. Due to its small population size (10 features in a population), any particular feature is less likely to be selected repeatedly by random sampling.

**Table 3:** Ten features (*m*/*z *ratio) selected by Genetic algorithm coupled with LDA in our 10 fold cross validation.

Round 1	Round 2	Round 3	Round 4	Round 5	Round 6	Round 7	Round 8	Round 9	Round 10
3385	4943.6	11555	1489.9	5939.7	1859.5	5098.2	5916	9835.1	5775.7
3304.8	5775.7	1125.2	11541	5209.5	2009.5	9578.9	2016.7	1857.2	3833.4
3186.7	4013.8	4943.6	1644	5822.1	2951.2	3760.5	3787.6	11940	3510.5
1858.7	3915.5	3383.7	4941.6	1063.5	11662	7553.5	5857.1	2756.1	1857.2
4256.8	1858	1528.6	5409.1	1644.6	11546	3540.1	3727.5	1808.1	11031
3790.7	1063.1	3959.6	1936.1	1859.5	9415.6	1860.2	1064	4532.7	5801.2
4941.6	1476.3	3726	2368.5	11463	7406.9	7966.1	5819.8	7931.2	4318.6
11027	3727.5	5829.1	3188	7592.8	6569.5	11394	9640.4	11477	11821
11426	11560	3188	1859.5	11472	1645.9	10183	1702.2	6511.9	5794.2
7085.3	2579.1	5949.2	3836.4	6509.3	1411.1	9575	6506.7	4941.6	9640.4

### Classification results and comparisons

After data dimension reduction using methods mentioned above, we tested and compared the performances of a single decision tree algorithm C4.5, six different decision tree-based classifier ensembles, and six different benchmark classification algorithms in a 10 fold cross validation framework. Default parameters were used in all algorithms without any fine-tuning of individual classifier, thus, allowing us to compare the performance of each algorithm. Because no particular classifier is preferred, potential misleading conclusion can be avoided.

Table 4 lists the average performances of all algorithms in a 10-fold cross validation using selected features from *t *test. In terms of accuracy, all classifier ensembles such as Bagging and Multiboost outperformed single decision tree (64% accurate) or at least had similar results. Moreover, the fact that single decision tree C4.5 has the best prediction sensitivity (0.99) and lowest specificity (0.21) indicates that this model was well adapted to only one class, thus failed to discriminate between both classes. The trade-off between prediction's sensitivity and specificity has been observed in many cases before [42] and was thought to result from the choice of threshold value chosen for making binary predictions. Similar trade-off effect in prediction is also associated with TP (true positive) and FP (false positive) rate.

**Table 4:** Classification results using features selected by Student *t *test.

Algorithm	Accuracy(%)	TP rate	FP rate	TN rate	FN rate	Sensitivity	Specificity	Precision	Fmeasure	RMSE
C4.5	0.6444	0.99	0.79	0.21	0.01	0.99	0.21	0.61	0.76	0.4687
Random Forest	0.6500	0.79	0.53	0.48	0.21	0.79	0.48	0.65	0.71	0.4569
Bagging	0.6833	0.78	0.44	0.56	0.22	0.78	0.56	0.69	0.73	0.4285
Logitboost	0.6889	0.83	0.49	0.51	0.17	0.83	0.51	0.69	0.75	0.4402
Stacking	0.6444	0.99	0.79	0.21	0.01	0.99	0.21	0.61	0.76	0.4761
Adaboost	0.6444	0.77	0.51	0.49	0.23	0.77	0.49	0.69	0.69	0.4412
Multiboost	0.6889	0.81	0.46	0.54	0.19	0.81	0.54	0.70	0.74	0.5175
Logistic	0.7500	0.79	0.30	0.70	0.21	0.79	0.70	0.78	0.78	0.4224
Naivebayes	0.6833	0.64	0.26	0.74	0.36	0.64	0.74	0.76	0.68	0.5289
Bayesnet	0.6722	0.63	0.28	0.73	0.37	0.63	0.73	0.74	0.67	0.5308
Neural Network	0.7000	0.70	0.30	0.70	0.30	0.70	0.70	0.75	0.72	0.4517
RBFnet	0.6722	0.76	0.44	0.56	0.24	0.76	0.56	0.69	0.71	0.4632
SVM	0.6944	0.71	0.33	0.68	0.29	0.71	0.68	0.74	0.71	0.5489

TP rate: True positive rate, FP rate: False positive rate, TN rate: True negative rate, FN rate: False negative rate, RMSE: Root Mean Squared Error. RBFnet: Radio Basis Function network, SVM: Support Vector Machine.

Besides accuracy, mean squared error of prediction (MSE) is another important measure of performance. MSE is the expected value of the square of "error" and consists of two components – prediction variance and the square of the prediction bias. In many contexts, variance and bias of a single classifier can be effectively reduced by constructing classifier ensemble such as Bagging and Adaboost [43,44]. Our results support this observation. For example, RMSE (Root Mean Squared Error) of single decision tree C4.5 is 0.4687, which is higher than those of Random Forest (0.4569), Bagging (0.4285), Logitboost (0.4402) and Adaboost (0.4412), but interestingly smaller compared to those of Stacked generalization (0.4761) and Multiboost (0.5175) (Table 4). Most of the benchmark algorithms have higher RMSE compared to either single decision tree or classifier ensembles.

Similarly, using our top 10 features selected from the Wilcoxon rank test (Table 5), the prediction accuracy (66.67%) of a single decision tree is lower than those of Random forest, Logitboost and Multiboost, but similar to those from Stacked generalization and Bagging. The trade-off between prediction's sensitivity and specificity still exist for C4.5 and other classifiers. This effect is even more obvious for Stacked generalization. In general, the classification results from *t *test and Wicoxon rank test have no significant difference, indicating that both feature selection methods work equally well in this context.

**Table 5:** Classification results using features selected by Wilcoxon rank test.

Algorithm	Accuracy(%)	TP rate	FP rate	TN rate	FN rate	Sensitivity	Specificity	Precision	Fmeasure	RMSE
C4.5	0.6667	0.90	0.63	0.38	0.10	0.90	0.38	0.65	0.75	0.4683
Random Forest	0.7000	0.79	0.41	0.59	0.21	0.79	0.59	0.71	0.74	0.4401
Bagging	0.6667	0.68	0.35	0.65	0.32	0.68	0.65	0.72	0.69	0.4484
Logitboost	0.6833	0.76	0.41	0.59	0.24	0.76	0.59	0.70	0.73	0.4499
Stacking	0.6667	0.93	0.66	0.34	0.07	0.93	0.34	0.64	0.76	0.4639
Adaboost	0.6611	0.76	0.46	0.54	0.24	0.76	0.54	0.68	0.71	0.4805
Multiboost	0.7000	0.73	0.34	0.66	0.27	0.73	0.66	0.74	0.73	0.5187
Logistic	0.6556	0.77	0.49	0.51	0.23	0.77	0.51	0.67	0.71	0.4362
Naivebayes	0.6944	0.70	0.31	0.69	0.30	0.70	0.69	0.77	0.72	0.4969
Bayesnet	0.6778	0.73	0.39	0.61	0.27	0.73	0.61	0.71	0.71	0.5232
Neural Network	0.6778	0.66	0.30	0.70	0.34	0.66	0.70	0.73	0.68	0.4606
RBFnet	0.5944	0.74	0.59	0.41	0.26	0.74	0.41	0.62	0.67	0.4556
SVM	0.6611	0.71	0.40	0.60	0.29	0.71	0.60	0.71	0.70	0.5760

In contrast, features selected from the genetic algorithm show large variations compared to those features selected from *t *test and Wilcoxon rank test. However, it is unclear whether the classification results using genetic algorithm also vary significantly. In our study, we observed a similar pattern in prediction accuracy and RMSE value for genetic algorithm (Table 6). Classifier ensembles usually outperform a single decision tree. For example, a single decision tree has the lowest prediction accuracy (59%) compared to other classifier ensembles. Interestingly, the general performances of classifiers based on the feature selection method of genetic algorithm are considerably lower than those from *t *test and Wilcoxon rank test, possibly because the heuristic nature of wrapper method can not guarantee that the best features will be selected.

**Table 6:** Classification results using features selected by genetic algorithm.

Algorithm	Accuracy(%)	TP rate	FP rate	TN rate	FN rate	Sensitivity	Specificity	Precision	Fmeasure	RMSE
C4.5	0.5944	0.61	0.43	0.58	0.39	0.61	0.58	0.64	0.62	0.5718
Random Forest	0.6000	0.71	0.54	0.46	0.29	0.71	0.46	0.63	0.66	0.5047
Bagging	0.6111	0.64	0.43	0.58	0.36	0.64	0.58	0.66	0.65	0.4965
Logitboost	0.6167	0.68	0.46	0.54	0.32	0.68	0.54	0.65	0.66	0.5153
Stacking	0.6056	0.66	0.46	0.54	0.34	0.66	0.54	0.65	0.65	0.4892
Adaboost	0.6167	0.67	0.45	0.55	0.33	0.67	0.55	0.65	0.65	0.5960
Multiboost	0.6111	0.68	0.48	0.53	0.32	0.68	0.53	0.65	0.66	0.6147
Logistic	0.6056	0.67	0.48	0.53	0.33	0.67	0.53	0.63	0.65	0.5122
Naivebayes	0.6000	0.76	0.60	0.40	0.24	0.76	0.40	0.62	0.67	0.5251
Bayesnet	0.5611	0.73	0.65	0.35	0.27	0.73	0.35	0.59	0.65	0.5110
Neural Network	0.5944	0.61	0.43	0.58	0.39	0.61	0.58	0.65	0.62	0.5814
RBFnet	0.6000	0.69	0.51	0.49	0.31	0.69	0.49	0.63	0.65	0.5038
SVM	0.6333	0.72	0.48	0.53	0.28	0.72	0.53	0.66	0.68	0.5985

Recently, the area under ROC (Receiver Operating Characteristic) curve (AUG) has been widely used as a measure to compare the performance of different classifiers. Theoretically, AUG value equals the probability of correctly classified one pair of samples (each from one class). Therefore, one classifier is considered better if it has a larger area under the ROC curve compared to a different classifier. Thus, the AUG value under the ROC curve provides another measure of classifier performance. For example, the AUGs of classifiers using *t *test selected features are summarized in Table 7. Single decision tree C4.5 has the lowest AUG value (0.5625) while Random Forest has the largest AUG value (0.9375) among all classifier tested. These results strongly suggest the need to construct classifier ensembles to analyze proteomics data.

**Table 7:** AUG results of classifiers

Algorithm	AUG	Algorithm	AUG	Algorithm	AUG	Algorithm	AUG
C4.5	0.5625	Logitboost	0.8438	Bayes Net	0.8563	RBFnet	0.9
Random Forest	0.9375	Stacking	0.5625	Logistic	0.925	SVM	0.7
Random Tree	0.825	Adaboost	0.85	Neural Network	0.85		
Bagging	0.85	Multiboost	0.875	Naïve Bayes	0.8875		

## Discussion

Sensitive detection of clinically useful biomarkers and the building of a reliable predictor specific to pre-malignant pancreatic cancer will certainly aid the early detection of this deadly disease. Here, we propose the use of a more accurate decision tree-based classifier ensembles combined with feature selection methods to address some of the challenges facing current cancer proteomics data analysis. We are able to build a low bias and a low variance predictor using model-averaging method: classifier ensembles. This method greatly improves the accuracy of classification. Furthermore, the use of three feature selection methods have allowed us to select biomarkers that achieve the best classification performance and at the same time give us potential new insights into disease mechanism involved in cancer development.

Biological data sets generated from proteomics studies typically have a very high number of features compared to their small sample sizes. Many feature selection methods have been used in proteomic data analyses to reduce the high dimensionality of the dataset. These include methods such as information gain [37], Kolmogorov-Smirnov test [34] and random forest [35]. In our study, we used three different feature selection methods: *t *test, Wilcoxon rank test and genetic algorithm. These methods are derived from the two major schemas in feature selection, namely the filter and wrapper method [45]. Filter method is more efficient, reliable, and not subjected to any learning algorithm. However, this method considers each feature independently without regard to its relevance or the possibility that combination of features can improve classifier performance. In contrast, the Wrapper method chooses a particular learning algorithm as its performance guide to consider how useful some feature combinations are to the predictor. In genetic algorithm, the initial size of the population sampled from the whole dataset significantly affects the output result. Because of this, our repeated runs using genetic algorithm failed to yield similar results. The unreliability of genetic algorithm may limit its future utility in proteomics data analysis. Using the three methods mentioned earlier, we observed a generally consistent performance of all classifiers. Their accuracies range from 50% to 70%. Thus, feature selection methods used here are sufficiently robust for classification purpose.

Over the last two decades, intensive explorations of model-averaging methods for classification purposes produce a group of efficient decision tree-based classifier ensembles. In many different contexts, classifier ensembles outperform decision tree model and other single algorithms because of their superior ability to handle data variance. This is also demonstrated in our result. In all three feature selection method cases, classifier ensembles have better prediction accuracies. Meanwhile, many attempts were made to compare classifier ensemble techniques, but most of them only focused on the two most popular methods: Bagging and Adaboost. Although Stacked generalization, Multiboost and Logitboost have been proposed earlier, only recently these methods gained greater popularity in machine learning and bioinformatics community [46-48]. Until now, no direct comparisons of their performances were made. Our study represents the first attempt in this direction by considering them in the context of pancreatic cancer proteomics analysis.

In general, the performances of classifiers tested on the premalignant pancreatic cancer dataset are lower than we had expected, with the best prediction accuracy of 70% in a single run. There are two possible reasons for this. First, this proteomics dataset comes from mice with histologically confirmed premalignant PanIN but no evidence of invasive or metastatic disease [49]. Therefore, in the early developmental stage of pancreatic cancer, the levels of biomarkers may not exhibit significant differences between the normal and disease group. Secondly, we used the default parameters for all our classifiers without performing any fine-tuning. The advantage of doing this is that it can prevent the problem of "over-fitting" because the parameters we used are not adapted to a specific dataset, thus our method can be generalized to more datasets. The disadvantage of using the default parameters is that our result may not represent the best possible results.

## Conclusion

We presented a systematic machine learning method to analyze cancer proteomics data that utilized decision tree based classifier ensembles and three popular feature selection schemas in a cross validation framework. Our method includes three steps: preprocessing, feature selection and classification. The proposed method is general enough that it can be adapted to other proteomics data analysis problems. Our results show that classifier ensembles perform significantly better than single decision tree algorithm, highlighting the utility of classifier ensembles in future proteomics research. Additionally, biomarkers selected in this process may shed new lights on processes and mechanisms underpinning cancer development. Our study represents one of the first attempts to apply and compare decision tree based classifier ensembles in the context of cancer proteomics data analysis. Results presented here will open up other possibilities for further research.

## Methods

### Premalignant pancreatic cancer mass-spectrometry data

Pancreatic cancer peptide mass-spectrometry data was downloaded from the FDA-NCI Clinical Proteomics Program [50]. This dataset was generated from serums of 33 mice (5.5 ± 0.25 months) that carried low-level burdens of human pancreatic intraepithelial neoplasias (PanINs) cells that were induced by endogenous expression of *KRAS*^G12D^, and 39 age-matched control mice [49]. There are a total of 80 PanINs serum samples that are referred to as disease group and 101 control serum samples that are referred to as control group. For each serum sample, the data stream was binned using a fraction of 400 parts per million (ppm), thus condensed the data from 350,000 to 6771 data points. The *m*/*z *ratios range from 800 to 11992.91.

### Mass-spectrometry data preprocessing

In general, a typical mass-spectrometry data set contains several thousands of intensity measurements. Many factors such as system artifacts make mass-spectrometry data extremely noisy. Therefore, low-level preprocessing is critical to the success of data analysis [51]. Theoretically, observed mass spectra can be decomposed into three components [52]:

(1) *f*(*i*, *j*) = *b*(*i*, *j*) + *s*(*i*, *j*) + ε (*i*, *j*)

where *f*(*i*, *j*) is the observed value, *b*(*i*, *j*) is the baseline value, *s*(*i*, *j*) is the true signal and ε (*i*, *j*) is the noise for *i*th sample at *j*th *m*/*z *ratio. Baseline is considered to be the low frequency component of the observed signal and its variability arises from different sources such as sample ion dispensing, matrix chemical contamination and data collection. This problem is especially significant at low peak intensity because the noise to signal ratio is larger. Some of the baseline correction algorithms are summarized in [53]. To adjust for our baseline problem, we first estimated our baseline by segmenting the whole spectra into windows with a size of 200 *m*/*z *ratio intensities. We then used the mean value of these windows as the estimate of baseline value at that intensity [54]. Then a piecewise cubic interpolation method was used to perform regression, thereby avoiding the problem of sharp boundary. This procedure was applied to all spectrograms.

In mass spectrometry data, systematic differences between replicate experiments are often significant enough to prevent the drawing of any meaningful conclusion. To compensate for these systematic differences, we normalized the intensities of all spectrograms from the downloaded dataset. Many normalization methods developed for mass-spectrometry data are available and some of them have been successfully used in previous analysis [55-57]. Area under curves (AUC) which is defined as $\sum_{i=1}^{n}y_{i}$, where *y*_*i *_is the signal at *i*th *m/z *ratio, is used to measure the protein concentration in mass-spectrometry data. In this study, we standardized each spectrum based on the ratio of its area under curves (AUC) over the median calculated from all spectra [57]. Also, the maximum intensities from each spectrogram are rescaled to 100.

Raw mass spectrometry data typically contains signal and random noise introduced by factors such as instrument measurement error. Thus, it is important to reduce the noise in the data to improve the quality of the spectrograms. This enables feature selection schema to select significant features. We adopted a Gaussian kernel smoothing method to reduce the noise in our data. Assuming the signals are generated from a Gaussian distribution, we substitute each original data point value with a weighted average of all samples close to it. Each nearby data point × contributes according to its distance in a Gaussian form. Weights are determined by $w=e^{\left(-{\left(2dist/d\max\right)}^{2}\right)}$ where *dist *is the distance between this point and the center point and *dmax *is the maximum distance of all points and center point. In this work, we used bandwidth 10 to allow a reasonably large distribution such that there will be a five points on each side of the original data point.

### Feature selection

Two-sample student *t *test considers each feature independently. It assumes both groups of data values are distributed normally and have similar variances. Test statistics is calculated as follows:

(2) $$t=\frac{x_{d}-x_{c}}{\sqrt{\frac{{\mathrm{var}}_{d}}{n_{d}}+\frac{{\mathrm{var}}_{c}}{n_{c}}}}$$

Where *x*_*d *_and *x*_*c *_are the mean values of intensities from disease group and control group respectively. var_d _and var_c _are variances of two distributions. *n*_*d *_and *n*_*c *_are the numbers of instance in each distribution. This *t *value follows student *t *distribution with degree of freedom *n*_*d *_+ *n*_*c*_-2. The significance *p *value is calculated based on test statistics and *t *distribution.

Wilcoxon rank test is a nonparametric test which has no distribution assumption. All the data are ranked together based on their values. Then the ranks from one class are compared with those from the other class. The *U *statistics is calculated as:

(3) $$U_{d}=R_{d}-\frac{n_{d}(n_{d}+1)}{2}$$

where *n*_*d *_and *R*_*d *_are the size and sum of ranks in disease samples. An equally valid formula for U is to replace all values from control sample.

Although Wilcoxon rank test is robust against parameter variation and makes no distribution assumption, in situation such as proteomics data where the sample size is small, the *P *values calculated by Wilcoxon rank test tend to be higher. Therefore, it is not easy to detect statistically real difference. On the other hand, student *t *test can differentiate between these cases (if the distribution approximates Normal), thus is more powerful than nonparametric test in this context.

The wrapper method used in this study incorporate genetic algorithm as feature space search procedure. Genetic algorithm is a heuristic method. It adopts ideas from the field of evolutionary genetics, such as population, inheritance, cross-over, mutation and selection. Evolution starts from a group of randomly generated feature sets: the initial population. Individual's fitness (performance) is evaluated by a learning algorithm. The mutation process usually selects the individual with good fitness score from the parent population to form the next generation. Normally, the fitness of descendent population is better than their "parent" population. This process continues until the termination condition where fitness reaches maximum. Features in the final population will be reported. Factors such as the initial population, mutation rate, and local maximum can affect the performance of genetic algorithm. In general, genetic algorithm gives reasonably satisfactory result quickly.

### Decision tree ensemble algorithms

Decision tree is one of the most popular predictor used in machine learning community and is commonly used as a base learner in constructing classifier ensemble [58]. In decision tree algorithm, the approximated target function is represented as a tree-like structure. In general, it works by sorting down the tree branch from the root to some leaf nodes. Each internal node represents a specific test of instance attribute, and each branch represents one of the possible test results. The classical decision algorithm C4.5 [59] implements a top-down greedy search schema to search through all possible tree spaces. At each split, they try every possible feature to achieve maximum reduction of impurity. Decision tree is efficient, easy to interpret and robust but may suffer from low accuracy and high variance. Thus, many attempts were made to improve it using model averaging method.

Bagging (**B**ootstrap **Agg**regating) represents one of the first successful attempts to use model averaging method. It was originally proposed by Leo Breiman [60]. To build ensembles, Bagging repeatedly samples the training set data to form subset with replacement following a uniform probability distribution. Thus in each subset, one instance can appears more than once. One classifier is trained for each newly formed subset. The final classification result is determined by the unweighted votes of each classifier in the committee, thus aggregating all classifiers:

(4) $$H(x)=\arg\max\sum_{i=1}^{T}1(h_{i}(x)=y)$$

Where *H*(*x*) is the final committee vote result, *h*_i _is the result from individual classifier.

AdaBoost [61] is similar to Bagging in that both of them resample from the base of the training set. However, one of the major differences between them is that AdaBoost associates a different weight to each instance based on previous classification result. All instances are assigned equal weights at the beginning. After first round of classification, instances that are classified correctly will receive smaller weights in the next round. Instances that are incorrectly classified will have larger weights. By normalizing these weights to form another distribution, AdaBoost will sample from new distribution to train another classifier. The final result is based on weighted sum of all classifiers' results:

(5) $$H(x)=sign\left(\sum_{i=1}^{T}\alpha_{i}h_{i}(x)\right)$$

Where *H*(*x*) is the final prediction result, α_*i *_and *h*_*i *_are weight and result from individual predictors.

In the context of binary classification, the above Adaboost algorithm can be called discrete Adaboost. Friedman et al. [62] proved that Boosting algorithm is a stage-wise estimation procedure for fitting additive logistic regression model by minimizing an exponential criterion. This criterion is equivalent to a second order binomial log-likelihood criterion in the Taylor's series. Based on this discovery, they explored the possibility of using Bernoulli log-likelihood criterion, which in turn, called Logitboost. Friedman et al. showed that Logitboost could achieve equivalent level of performance compared to Adaboost [62] using a collection of datasets from the data repository located at University of California (Irvine).

Since the successful introduction of Bagging and Boosting algorithms, many investigators have tried to combine the power of variance reduction from Bagging and bias reduction from Adaboost. One attempt is Multiboost [63]. It tries to combine the benefits offered from both Bagging and Adaboost by exploring Wagging (**W**eight **Agg**regating, a variant of bagging), a set of committee formed by Adaboost. It also has an advantage in computation because these committees can learn in parallel. Wagging, which also repeatedly perturb the training data, does not sample the data to form smaller subsets. Instead, it adds noises to all weights such that it no longer assumes a uniform distribution. Thus, wagging take full advantage of the dataset without leaving any of the data unused. Using University of California (Irvine) repository datasets, Multiboost algorithm was frequently shown to achieve lower error than either Bagging or Boosting [63].

Random forest is another type of tree ensemble [64]. It can be considered as Bagging with random feature selection. In the forest, each tree is built using a bootstrap sample of the data. Candidate feature set is selected randomly at all tree splits. Randomness in the algorithm guarantees that low level of correlation between trees. In addition, each tree grows fully without any pruning. Thus, both variance and bias reduction can be achieved at the same time.

All the above algorithms have only one level in that all parallel trees take the original input data and provide one output prediction result. In contrast, Stacking [65] is a method that combines multiple level models for classification. Usually, there are two levels of classifiers. The first level classifiers are trained on the original input data, and their outputs are collected into a new dataset. This new dataset in turn serves as an input data for a second level learning algorithm that produce the final result. Many different combinations of level one and level two classifiers have been tested. Ting and Witten [66] showed that they can achieve the least error rate compared to other classifiers using output class probability together with least squares linear regression as their second level generalizer.

Also, bench mark algorithms such as Logistic Regression, Naïve Bayes, Bayes Net, Neural Network, Radial Basis Function Network and Support Vector Machine are also used in this study for the purpose of comparison.

### Classifier performance measurement

Ten fold cross validation is known to be a good estimator of classifier's performance. Ten percent of the data from the whole dataset are chosen randomly as test set while the remaining 90 percent are used as training set. This process is repeated 10 times and the average result is reported. In each run, prediction results can be classified into four groups: TP (true positive), FP (false positive), TN (true negative) and FN (false negative). Thus

Accuracy=nTPnTP+nFP+nTN+nFNTPrate=Sensitivity=Re⁡call=1−FNrate=nTPnTP+nFNFPrate=1−Specificity=1−TNrate=nFPnFP+nTNPr⁡ecision=nTPnTP+nFPFmeasure=2×Pr⁡ecision×Re⁡callPr⁡ecision+Re⁡call

And MSE (Mean Squared Error) can be decomposed as

(6) $$MSE(\hat{\theta })=Var(\hat{\theta })+{\left(Bias(\hat{\theta },\theta )\right)}^{2}$$

Where $\hat{\theta }$ is the estimator of parameter *θ*.

There is always a trade-off between sensitivity and specificity because of the different threshold values used in binary prediction. Thus, ROC (Receiver Operating Characteristic) curve is used to plot true/false positive rates or sensitivity/1-specificity for different thresholds. The area under the ROC curve (AUG) equals the probability of correctly classified one pair of samples, each one from a separate class. It has been used as an important measurement of classifier performance. A classifier is considered a preferred classifier compared to the other classifier if it has a larger AUG value. A random classifier has an area of approximately 0.5 under the ROC graph, whereas a perfect classifier has an area of 1.

Weka machine learning package was used in the simulation of classifier ensembles [67]. We also used Matlab statistics and bioinformatics toolbox in the data preprocessing and feature selections.

## Authors' contributions

GG conceived of the study and carried out the computational experiment, GG and GWW drafted the manuscript. All authors read and approved the final manuscript.
